# Effect of micro-osteoperforations on the rate of orthodontic tooth movement and expression of biomarkers: a randomized controlled clinical trial

**DOI:** 10.1590/2177-6709.27.1.e2219403.oar

**Published:** 2022-06-06

**Authors:** Pradeep RAGHAV, Amit Kumar KHERA, Preeti PREETI, Shalu JAIN, Stuti MOHAN, Anurag TIWARI

**Affiliations:** 1 Swami Vivekanand Subharti University Subharti, Dental College, Department of Orthodontics (Meerut, India).

**Keywords:** Micro-osteoperforation, Accelerating orthodontic tooth movement, Biomarker activity

## Abstract

**Introduction:**

Micro-osteoperforation is a minimally invasive technique that has been used to accelerate orthodontic tooth movement and reduce treatment duration. However, literature presents conflicting reports about this technique.

**Objective:**

To evaluate the effectiveness of micro-osteoperforations on the rate of canine retraction and expression of biomarkers in gingival crevicular fluid (GCF).

**Methods:**

This was a randomized clinical trial with split-mouth study design. Thirty adult subjects with age above 18 years (20.32 ± 1.96) who required fixed orthodontic treatment and extraction of maxillary first premolars were enrolled and randomly allocated to either the experimental or control group. Randomization was performed by block randomization method, with a 1:1 allocation ratio. The experimental group received three micro-ostoperforations (MOPs) distal to maxillary canine, using the Lance pilot drill. The retraction of maxillary canine was performed with NiTi coil-spring (150g) in both experimental and control groups. The primary outcome was the evaluation of canine retraction rate, measured on study models from the baseline to 16 weeks of canine retraction. Secondary outcomes were the estimation of alkaline and acid phosphates activity in GCF at 0, 1, 2, 3, and 4 weeks.

**Results:**

There was a statistically significant difference in the rate of canine retraction only after the first 4 weeks. Subsequently there was no statistically significant difference from the eighth to the sixteenth weeks between MOPs and control group. There was a statistically significant difference in alkaline and acid phosphates activity in GCF between MOPs and control groups during the initial 4 weeks of canine retraction.

**Conclusion:**

Micro-ostoperforation increased the rate of tooth movement only for the first 4 weeks; thereafter, no effect was observed on the rate of canine retraction during 8, 12 and 16 weeks. A marked increase in biomarker activity in the first month was observed.

## RESUMO

**Introdução:**

As micro-osteoperfurações (MOPs) são uma técnica minimamente invasiva que tem sido utilizada para acelerar a movimentação dentária ortodôntica e reduzir o tempo de tratamento. No entanto, existem relatos conflitantes sobre o uso dessa técnica.

**Objetivo:**

Avaliar a eficácia das MOPs em acelerar a taxa do movimento de retração de caninos e na expressão de biomarcadores no fluido crevicular gengival (FCG).

**Métodos:**

Esse foi um ensaio clínico randomizado com desenho de estudo do tipo boca dividida. Trinta indivíduos adultos com idade acima de 18 anos (20,32 ± 1,96 anos) que necessitavam de tratamento ortodôntico fixo e extração de primeiros pré-molares superiores foram incluídos e aleatoriamente alocados para o grupo experimental ou grupo controle. A randomização foi realizada pelo método de randomização em bloco, com proporção de alocação de 1:1. O grupo experimental recebeu três MOPs distais ao canino superior, utilizando uma broca piloto em formato de lança. A retração do canino superior foi realizada com mola helicoidal de NiTi (150g) nos dois grupos, experimental e controle. O desfecho primário foi a avaliação da taxa de retração dos caninos, medida em modelos de estudo do início da retração até 16 semanas depois. O desfecho secundário foi a estimativa da atividade da fosfatase alcalina e ácida no FCG após 0, 1, 2, 3 e 4 semanas.

**Resultados:**

Houve uma diferença estatisticamente significativa na taxa de retração dos caninos somente após as quatro primeiras semanas. Após isso, não houve diferença estatisticamente significativa entre os grupos experimental e controle entre a oitava e a décima sexta semanas. Houve uma diferença estatisticamente significativa na atividade da fosfatase alcaline e ácida no FCG entre os grupos experimental e controle durante as quatro primeiras semanas de retração dos caninos.

**Conclusão:**

As micro-osteoperfurações aumentaram a taxa de movimentação dentária apenas nas primeiras quatro semanas; depois disso, nenhum efeito foi observado na taxa de retração dos caninos após 8, 12 e 16 semanas. Houve aumento considerável na atividade do biomarcador no primeiro mês.

## INTRODUCTION

Over the past decade, accelerated orthodontic movement has become an encouraging area of research in the orthodontic field. Several techniques have claimed to improve orthodontic treatment efficiency, by reducing treatment duration in complex adult treatment.^1^ ^,^ ^2^ Current research indicates that the most effective methods for the acceleration of tooth movement are the surgical approaches, including distraction osteogenesis, corticotomy, osteotomy, and piezocision technique. However, it is assumed that the surgical approaches have not been widely employed, due to the aggressiveness and associated complications.^3^ ^-^ ^6^

Recently, less invasive and controlled micro-trauma through micro-osteoperforations (MOPs) have shown promising outcomes, with considerably less risk of surgical associated complications. MOPs accelerate tooth movement by augmenting the expression of inflammatory markers.^7^ ^,^ ^8^ During an inflammatory response, a repertoire of inflammatory mediators is activated, leading to the recruitment of inflammatory cells and osteoclast precursors into the inflamed area; which directly or indirectly activate the Receptor Activator of Nuclear factor-kB (RANK) and Receptor Activator of Nuclear factor-kB Ligand (RANKL) pathway, leading to osteoclastic differentiation and activation. This evidence indicates that the MOPs increase the catabolic and anabolic activities, thus reducing tooth movement resistance. These catabolic and anabolic activities can be measured by the expression of bone resorption and bone formation biomarkers in gingival crevicular fluid (GCF).^9^

Teixeira et al.^8^ conducted a study on rats and stated that minimum cortical perforations increased the inflammation and enhanced tooth movement. Recently, many studies have been conducted to evaluate the effect of MOPs on the rate of tooth movement.^10^ ^-^ ^14^ Some of these studies have shown an increase in the rate of tooth movement in the experimental group of more than 2 folds, when compared to the control group.^10^ ^,^ ^11^ However, contradictory results have also been reported by several studies.^12^ ^,^ ^13^ According to a Cochrane review,^14^ most of the randomized clinical trials presented small sample size and unclear risk of bias. A recently conducted meta-analysis indicated that there was a statistically significant difference in the rate of canine retraction after performing MOPs; however, clinically, it failed to show substantial outcomes.^15^

Up to the present date, several articles have been published on accelerated orthodontics, but there is a lack of information on the relationship between bone catabolic and anabolic biomarkers. Ferguson et al.^9^ conducted a systematic review to evaluate the effect of various surgical accelerating techniques on the expression of biomarkers, and found that most of the studies were done using animals. Assessing a human sample, only the report published by Alikhani et al.^4^ was found. The authors evaluated the effect of MOPs on the expression of inflammatory markers, and found that there was 2.3-fold increased rate of canine retraction, with increased expression of cytokines. The study comprised a small sample size, and follow-up was done for only 28 days; moreover, a possible conflict of interest should be discussed, because they used commercially available appliances, and randomization and allocation concealment was unreported. In addition, they used lateral incisor as a reference point to measure canine retraction, which is considered an unstable point, and used 0.016*x*0.022-in stainless wire in 0.022-in slots for canine retraction, a procedure that allows more tipping movement and could give a false perception of tooth movement acceleration. All these shortcomings signify that there is an urgent need for high-quality randomized controlled clinical trials that helps proving the effectiveness of MOPs and its correlation with the expression of bone biomarkers. 

Therefore, the present study is, as far as we know, the second study done with human sample, with a follow-up of 16 weeks, to evaluate the effect of MOPs on the rate of canine retraction and its correlation with expression of biomarkers in GCF. The null hypothesis tested was that there is no difference in the rate of canine retraction and the level of the biomarkers in the GCF between control and micro-osteoperforated group.

### SPECIFIC OBJECTIVES OR HYPOTHESES

The objectives of the present study were:

» Evaluate the effect of MOPs on the rate of canine retraction for a period of 16 weeks.
» Evaluate the changes in the level of biomarkers in the GCF.

## MATERIAL AND METHODS

### TRIAL DESIGN

The present study was a single-center randomized controlled clinical trial using a split-mouth design, with 1:1 allocation. No changes were made after the initial trial.

### PARTICIPANTS, ELIGIBILITY CRITERIA AND SETTINGS

Ethical approval was obtained from ethical reviewer board of the institute at Swami Vivekanand Subharti University (Meerut/India). The trial was also registered at ICMR with CTRI number 01516450. Subjects were screened from the department of orthodontics, Subharti Dental College, between April 2018 and October 2018. A sample of 30 subjects met the inclusion criteria summarized in Table 1. A detailed medical history was recorded for each patient, followed by a detailed clinical examination. Informed written consent was taken from patients or parents/legal guardians, after informing the study procedures.

**Table 1:** Inclusion and exclusion criteria for the study.

Inclusion criteria	Exclusion criteria
Male or female	Long term use of antibiotics, steroids, anti-inflammatory drugs
Age range: 17-30 years	Prominent canine roots (in labial cortical bone)
Angles’ Class I bimaxillary, Class II div. 1 malocclusion (ANB < 5º)	Extreme skeletal Class II malocclusion (ANB > 5º)
No systemic disease	
No radiographic evidence of bone loss	
No history of periodontal therapy	
No smoking	
No gingivitis or untreated caries	
Gingival index <1	
Plaque index <1	

### SAMPLE SIZE CALCULATION

The sample size was calculated based on a type I error frequency of 5%. Power analysis with G*Power software showed that 27 subjects per group would be needed for a statistical power of more than 80% to detect a significant difference, with 0.66 effect size and 0.05 as the significance level.

### RANDOMIZATION

MOPs were performed on a total of 30 patients, either in the right or left side. The side of the maxillary arch where MOPs were performed was considered as the experimental side, while the other, as the control side. To remove selection bias, surgical intervention was randomly allocated to the right or left side. The randomization was achieved by block randomization method, with a block size of 6. The concealment of allocation sides was made using sealed opaque envelopes, which were shuffled by a faculty member. Each subject was provided with an envelope and sent to the operator (oral surgeon) who was performing the MOPs. 

### BLINDING

Blinding of either patients or orthodontist was not possible; therefore, blinding was implemented at the measurement level. All the study models were coded. Measurements and data collection were performed by other investigator who was blinded to which side MOPs were performed.

### INTERVENTION

Extraction of first premolars were performed 4 months before initiation of canine retraction or at the beginning of treatment, to avoid a confounding factor, because surgical trauma from extraction can also initiate RAP phenomenon. All subjects were bonded with 0.022 x 0.028-in MBT prescription appliance (3M Unitek, Monrovia, CA, USA). Leveling and alignment was initiated with nickel-titanium (NiTi) archwires, and continued until 0.019 x 0.025-in stainless steel wire. This wire was then kept in the bracket slot for 4 weeks, to allow passive contact before the initiation of canine retraction. This care was done to reduce the bias caused by the binding of wire in bracket slot. To further reduce the friction during canine retraction, stainless steel ligature wire was ligated loosely to the brackets. Before retraction start, canine root prominence was checked by a simple palpation method. If the canine root prominence was considered too evident, the subject was removed from the study or the root was torqued to move into the cancellous bone. Nance palatal button was used in all the subjects to reinforce the anchorage. Before performing MOPs, soft tissue thickness was analyzed using William’s probe.

**Figure 1:**
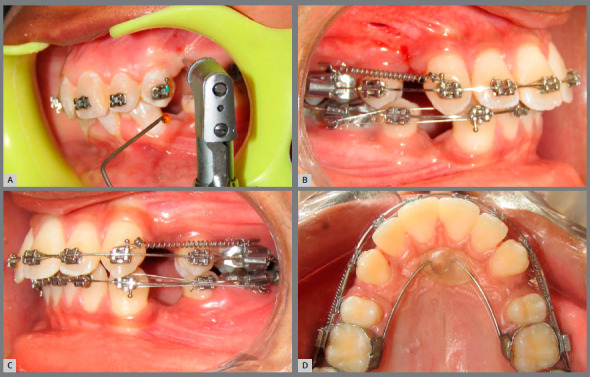
A) MOPs performed at distal to the canine, in experimental group. B) Canine retraction by NiTi coil spring in experimental group. C) Canine retraction by NiTi coil spring in control group. D) Occlusal view during canine retraction.

Strong topical anesthesia (Lidocaine Topical Aerosol USP 15% W/W) was applied for MOPs. After anesthesia was achieved, a Lance Pilot Drill (Alpha-Bio Tec, Simplantology Alpha Bio Tec LTD) was used to perform three perforations distal to canine root, with a perforation width and depth of 2 and 5 mm, respectively, under copious saline irrigation. Canine retraction was done on 0.019 x 0.025-in stainless steel wire with NiTi closed coil spring (GAC international), and a force of 150 g was used for retraction (Fig 1). The NiTi closed coil spring was attached from the canine power arm to the hook of a molar tube. At each appointment, a Dontrix gauge was used to measure the retraction force. If the force level was found to be less than 150 g, then NiTi coil spring was activated to maintain the force level. The bite contact was raised in those subjects in which occlusal interferences were present. 

### OUTCOMES (PRIMARY AND SECONDARY)

#### Primary outcome

**Figure 2:**
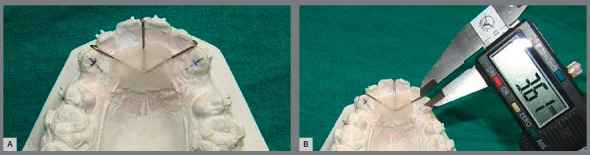
A) Palatal plug on study model. B) Measurement of canine retraction by digital caliper.

The rate of canine retraction was assessed as the primary outcome of the study. To monitor the rate of canine movement, alginate impressions were made before the canine retraction (T_0_) and after four weeks (T_1_), eight weeks (T_2_), twelve weeks (T_3_) and sixteen weeks (T_4_); and study models were fabricated with Type-II dental stone. For the measurement of canine retraction on the study model, the method used by Loztof et al.^16^ was applied, with a slight modification. In our method, an acrylic palatal plug with reference wires was fabricated on the first study model (T_0_) over the Nance palatal button used for anchorage control, and was stabilized with pinheads on anterior teeth. Reference stainless steel wires (0.9 mm) were placed mesial to canine on both sides, and the terminal ends were embedded in the acrylic plug. A long axis of canine was drawn from cusp tip to cervical end, and the midpoint of this axis was marked. The base value was set by measuring the distance from this midpoint to the mesial reference wire on the first model (T_0_) (Fig 2). The plug was then transferred to the subsequent study models (T_1_, T_2_, T_3_ and T_4_), and the distance that the canine has moved at every 4-week interval was measured. The palatal plug was considered as the reference device for all the study models of the same patient. All measurements were recorded by other blinded investigator, using a digital vernier caliper (Mitutoyo Corporation,Kawasaki, Japan) with accuracy of 0.01mm. During measurements, the caliper was oriented parallel to the long axis of canine and reference wire. At each visit, the force produced by the coil spring was checked and recalibrated. The appliance was examined for any breakage or deformation.

#### Secondary outcome

To evaluate the level of the inflammatory response, GCF samples were collected from each subject before MOPs and at 1, 2, 3, and 4-weeks after MOPs, between 10 AM and 12 PM. Supragingival plaque was removed and cotton rolls were used to isolate the regions. The samples were obtained from the distobuccal and mesiobuccal crevice of the maxillary canine of both right and left sides. GCF samples were collected with endodontic paper points (size 30), inserted 1 mm below the gingival margin, for 60 seconds. Immediately, the three dipped paper points (per site) were collected and diluted in 2 mL Eppendorf tube containing 200 µL of buffered saline. Then, this solution was centrifuged for 2 min at 2000 rpm, to elude the GCF components completely. Finally, the paper points were removed and the supernatant was stored at - 37ºC. Alkaline and acid phosphatase biomarkers in GCF were analyzed by spectrophotometer at all the time intervals, for both experimental and control groups.

### STATISTICAL ANALYSIS

Both intraobserver and interobservers errors were evaluated. For the assessment of intraobserver error, a sample of 15 randomly selected study models was measured by the same investigator after three weeks, to verify the reliability of the measurements. For interobservers error, a second investigator measured the same set of models twice; the mean values of the two measurements by each investigator were compared. Intraclass Correlation Coefficient (ICC) was used to assess intraobserver accuracy and reliability. ICC value was 0.9, which is in the range of excellent measurement agreement. The data were subjected to the Statistical Package for the Social Sciences (SPSS 22.0 version) for analysis. Shapiro-wilk test for normality was used to choose parametric or non-parametric test. All tested variables were normally distributed, hence independent *t*-test was used to compare the rate of canine retraction and level of biomarkers in GCF between the MOPs and control group. Values were considered significant when less than or equal to 0.05. 

## RESULTS

**Figure 3:**
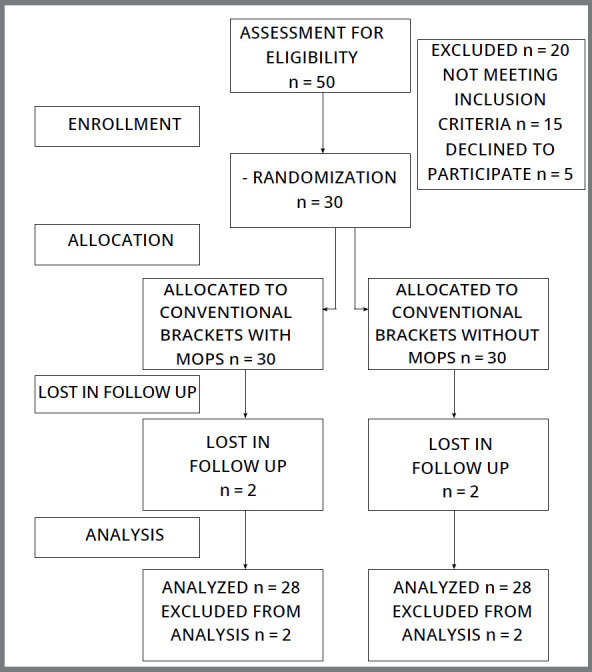
CONSORT flow chart.

Two subjects were excluded from the study due to irregular follow-up. Data from 28 subjects were analyzed (Fig 3). Subjects data including gender, age, amount of extraction space, and cephalometric analysis are listed in Table 2. Subject’s age ranged from 17 to 30 years (20.26 ± 2.13). No bracket failure was observed during canine retraction. The bite was raised in 6 subjects with glass-ionomer cement, to remove the occlusal interferences. No negative outcome was reported by any subject during the trial.

**Table 2:** Baseline data of subjects.

	n (%)	Mean ± SD
Age		20.26 ± 2.13
Sex		
Male	12 (40%)	
Female	18 (60%)	
SNA (degrees)		81.24 ± 2.89
SNB (degrees)		77.0 ± 2.56
ANB (degrees)		4.1 ± 3.16
SN.MP (degrees)		27.6 ± 3.45
GoGn.SN (degrees)		28.327.6 ± 3.23
U1.SN (degrees)		107.4 ± 3.45
IMPA (degrees)		98.34 ± 5.45
Pre-retraction maxillary extraction space		
Right side		6.01 ± 0.68
Left side		6.03 ± 0.54

### PRIMARY OUTCOME

The rate of canine retraction was measured after 4, 8, 12 and 16 weeks. The mean rate of space closure was 1.11 mm in the experimental group (MOPs) and 0.82 mm in the control group at the fourth week. The experimental group showed significantly greater canine movement when compared to the control group (T_1_, *p*< 0.05). In the remaining periods (8, 12 and 16 weeks), however, there was no statistically significant difference in the rate of canine retraction between experimental and control groups (*p*> 0.05, Table 3).

**Table 3:** Comparative study of mean rate of canine retraction at every four weeks between experimental and control groups (independent *t*-test).

Time intervals	Experimental Mean ± SD	Control Mean ± SD	***p-*value**
T1 - T0	1.1164 ± 0.486	0.8224 ± 0.423	0.0272 (S)
T2 - T1	0.896 ± 0.5110	1.0428 ± 0.6202	0.3754 (NS)
T3 - T2	1.1068 ± 0.5878	1.1548 ± 0.6314	0.7864 (NS)
T4 - T3	0.9996 ± 0.4128	0.9332 ± 0.5590	0.6420 (NS)

p ≤ 0.05 is significant (S), p > 0.05 is non-significant (NS).

T0 = Baseline, T1 = 4 weeks, T2 = 8 weeks, T3 = 12 weeks, T4 = 16 weeks.

### SECONDARY OUTCOME

**Figure 4:**
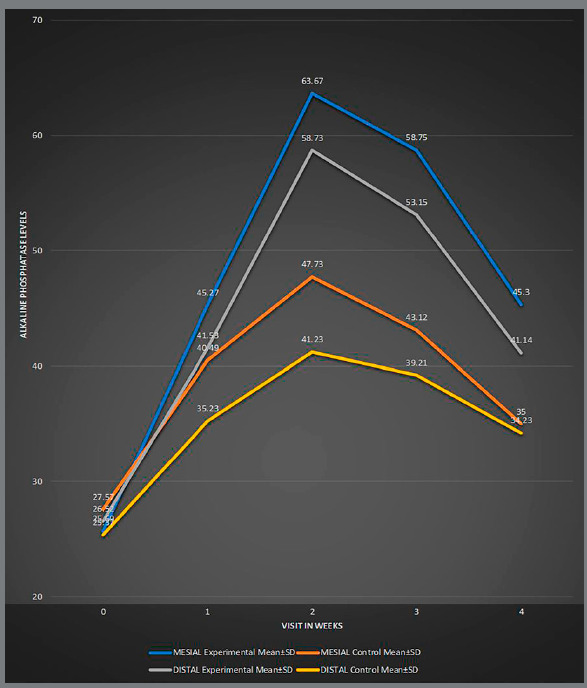
Comparison between the mesial and distal sides of MOPs and control groups at different time intervals, for alkaline phosphatase level.

**Figure 5:**
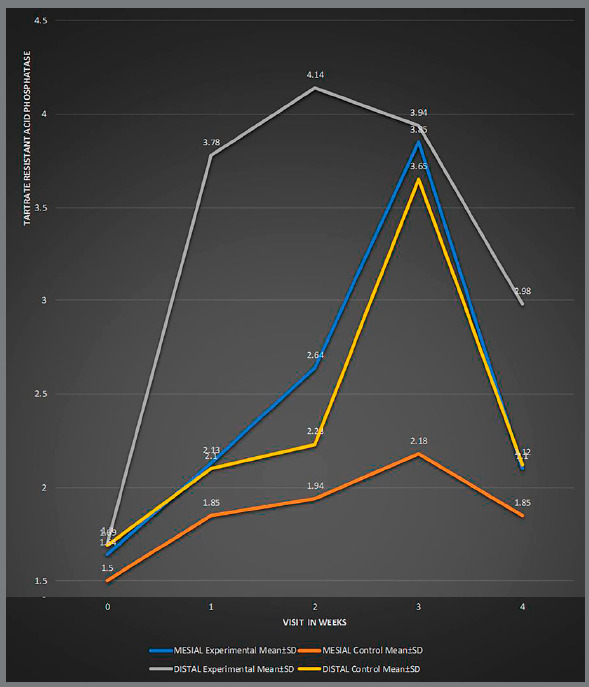
Comparison between the mesial and distal sides of MOPS and control groups at different time intervals, for tartrate resistant acid phosphatase.

There was a statistically significant difference in the level of alkaline phosphatase (ALP; Table 4, Fig 4) and acid phosphatase (TRAP; Table 5, Fig 5), on both mesial and distal sides, between experimental and control groups at different time intervals. The level of alkaline phosphatase was found significantly higher on the mesial side, while the level of acid phosphatase was found significantly higher on the distal side. The peak of ALP level occurred at the second week on mesial and distal sides, in both MOPs and control group. A marked ALP activity increase occurred in MOPs group after two, three and fours weeks, when compared to the control. The peak of TRAP occurred on distal side of canine (tension side) at the second week in MOPs group, and at the third week in control group. The level of TRAP was significantly higher in MOPs group, when compared to control group.

**Table 4:** Comparison between the mesial and distal sides of MOPs and control groups at different time intervals for alkaline phosphatase level.

Visit in weeks	Mesial	Mesial	*p-* **value**	Distal	Distal	*p-* **value**
	Experimental Mean ± SD (U/mg)	Control Mean ± SD (U/mg)		Experimental Mean ± SD (U/mg)	Control Mean ± SD (U/mg)	
0	25.69 ± 5.12	27.57 ± 4.12	0.1135 (NS)	26.52 ± 5.12	25.37 ± 4.12	0.0941 (NS)
1	45.27 ± 6.12	40.49 ± 5.7	0.1021 (NS)	41.53 ± 6.12	35.23 ± 5.12	0.1009 (NS)
2	63.67 ± 7.83	47.73 ± 6.12	0.0003 (S)	58.73 ± 6.12	41.23 ± 6.12	0.0000 (S)
3	58.75 ± 6.12	43.12 ± 5.13	0.0002 (S)	53.15 ± 5.12	39.21 ± 5.12	0.0000 (S)
4	45.30 ± 5.13	35.00 ± 4.12	0.0001 (S)	41.14 ± 4.12	34.23 ± 4.12	0.0003 (S)

p ≤ 0.05 is significant (S), p > 0.05 is non-significant (NS).

**Table 5:** Comparison between the mesial and distal sides of MOPs and control groups at different time Intervals, for tartrate resistant acid phosphatase.

Visit in weeks	Mesial	Mesial	*p-* **value**	Distal	Distal	*p-* **value**
	Experimental Mean ± SD (U/mg)	Control Mean ± SD (U/mg)		Experimental Mean ± SD (U/mg)	Control Mean ± SD (U/mg)	
0	1.64 ± 0.88	1.50 ± 0.94	0.1328 (NS)	1.70 ± 0.84	1.69 ± 0.92	0.1154 (NS)
1	2.13 ± 0.73	1.85 ± 1.15	0.1028 (NS)	3.78 ± 1.23	2.10 ± 1.10	0.0029 (S)
2	2.64 ± 0.94	1.94 ± 0.86	0.1008 (NS)	4.14 ± 0.96	2.23 ± 0.92	0.0008 (S)
3	3.85 ± 1.16	2.18 ± 1.61	0.1018 (NS)	3.94 ± 1.01	3.65 ± 0.84	0.1012 (NS)
4	2.10 ± 1.12	1.85 ± 1.85	0.1009 (NS)	2.98 ± 0.96	2.12 ± 0.89	0.1018 (NS)

p ≤ 0.05 is significant (S), p > 0.05 is non-significant (NS).

## DISCUSSION

The principle of micro-osteoperforations is a regional controlled micro-trauma, causing localized osteopenia and initiating a cascade of inflammatory reactions, ultimately recruiting inflammatory cells and osteoclast precursors from the periodontal ligaments (PDL) extra-vascular space. Thus, even shallow perforations increase the rate of tooth movement.^17^ ^-^ ^21^ Many confounding factors can affect the rate of tooth movement, such as the amount and aggressiveness of trauma, occlusal interferences, age, and type of tooth movement, oral hygiene, systemic diseases, and patient compliance. Some studies^22^ ^,^ ^23^ have shown that age plays an important role in tooth movement, and this effect is related to bone density and osteoclast recruitment or activation. Nimeri et al.^19^ has also documented that RANKL:OPG ratio is proportional to age, which affects the rate of bone remodeling and tooth movement. To minimize the effect of age, only adult subjects (age >17 years) were included in this study. Strict discipline and clear instructions were given to maintain excellent oral hygiene. Occlusal interferences in the path of canine retraction were removed by raising the bite, when necessary. According to Yang et al.^24^, maximum stress during the canine retraction was distributed on the cervix at distolabial side, and circumscribed cut reduced bone resistance. Based on their concept, in this study, three MOPs were performed only distal to canine at equal distance along the root length, to reduce the resistance offered by alveolar crest. Wilcko et al.^18^ stated that after MOPs, maximum effect of RAP phenomenon is observed for 2-3 months.^16^ Therefore, the effects of MOPs in the present study were investigated over 16 weeks. A Lance pilot drill was used to perform the MOPs, which is routinely used for implant placement. Tsai et al.^25^ showed positive results using a round bur with a low-speed handpiece. Similarly, Cheung et al.^20^ used mini-implant facilitated MOPs and showed 1.86-fold increase in the rate of tooth movement.^19^ Since the purpose of MOPs is to perforate only the alveolar bone, a Lance pilot drill was used, because it has sharp edges, calibrated length and is designed to cut the bone effectively. To effectively perforate the cancellous bone, the perforation depth was kept at least 5 mm, because average gingival thickness is 2 to 3 mm, and average cortical bone thickness is 1.5 to 2.0 mm. Although the thickness of attached gingival tissue may vary from patient to patient, for those patients in which attached gingival thickness was more than average, the perforation depth was increased to reach the cancellous bone. After MOPs, a force of 150 g was used for canine retraction, as recommended by Samuels et al.^26^

In this trial, the accelerating effect of MOPs was observed only during the first four weeks. In this period, the rate of canine retraction was increased by 1.3 fold (34%), when compared to control side. This evidence is similar to other studies^4^ ^,^ ^10^ ^,^ ^27^ that found increased rate of canine retraction by more than 2 folds. In the present study, the 8, 12 and 16 weeks evaluations showed no significant difference between experimental and control groups. These results were also in agreement with Alkabsi et al.^12^ Similar findings were observed in the meta-analysis conducted by Shahabee et al.^15^, who concluded that there was an increase in the rate of canine retraction after MOPs; however, clinically not very substantial. Blasi and Pavlin^28^ suggested that frequent use of MOP can sustain higher levels of inflammatory markers, leading to an increased rate of tooth movement. However, Sivarajan et al.^29^ stated that no significant benefit was observed with repeated use of MOP. In their study, after sixteen weeks, they found that there was higher tooth movement in the group receiving MOPs only twice (every eight weeks), as compared with the groups that underwent MOPs three or four times.

**Figure 6:**
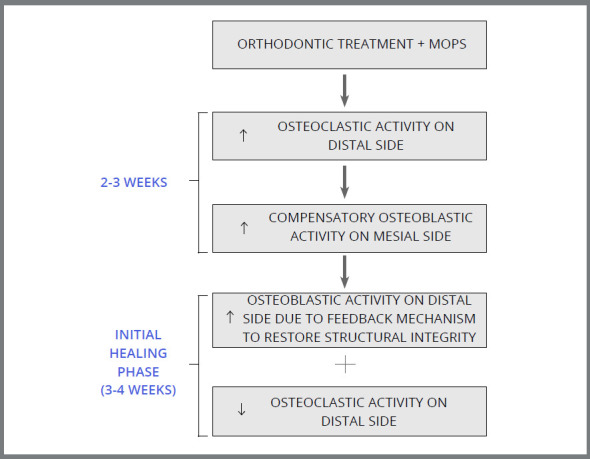
Cellular activity after micro-osteoperforation.

The secondary outcome of the present study was to correlate the rate of tooth movement with the expression of biomarkers after MOPs. The systematic review conducted by Kapoor et al.^30^ showed that there was a positive correlation between the levels of ALP and TRAP in the GCF and the velocity of orthodontic tooth movement, in a time-dependent manner. Many histological and human studies states that the maximum changes in the concentration of inflammatory markers occurred during the first four weeks after the application of orthodontic force or surgical injury.^4^ ^,^ ^31^ ^-^ ^33^ Therefore, in the present study, the evaluation of biomarkers was limited to four weeks. The present study showed that the peak of TRAP occurred on distal side of canine (tension side) at the second week in MOPs group, and occurred at the third week in control group. The level of TRAP was also significantly higher in MOPS group, when compared to control group, clearly indicating that there was intensified and early onset of osteoclastic activity on distal side in MOPs group, when compared to control group. In the fourth week, however, there was no significant difference in TRAP level between MOPs and control groups. The peak of ALP level occurred at the second week on mesial and distal sides in both MOPs and control groups, but there was a markedly increased ALP activity in MOPs group at the second, third and fourth weeks, when compared to control group. These results indicate that there was increased osteoblastic activity at the third and fourth week on mesial side (tension side), as a compensatory mechanism to increased osteoclastic activity (occurring in the initial two weeks on distal side). Increased osteoblastic activity on distal side (compression side) was due to homeostatic mechanism induced by micro-perforations. These biomarker activity shows that increased osteoclastic activity persist only for the first three weeks after the increased osteoblastic activity (Fig 6). These results directly correlate, and may be the reason for the faster tooth movement seen after four weeks. Consistently, Alikhani et al.^4^ and Ferguson et al.^9^ concluded that bone biomarkers such as TRAP and TNF-α increased during accelerated tooth movement, when compared to the control group, which further corroborates the result of the present study. Due to increased osteoblastic activity after four weeks, we did not find any significant increase in the rate of canine retraction after eight, twelve, and sixteen weeks.

The results of the present study, as well as other studies, clearly indicates that MOPs accelerate the orthodontic tooth movement until four to five weeks after induction of trauma. The reason behind the accelerating effect of MOPs might be that microtrauma induces alveolar bone inflammation, which leads to an increase in cellular activity, thereby increasing the bone turnover rate, causing a decrease in bone density, thus increasing orthodontic tooth movement. Thereafter, as the healing process progresses, bone remodeling returns to its initial pace and there is regaining of the bone density to the pre-MOPs level. 

The limitations of this study are the following: (1) GCF samples were evaluated only for a 1-month follow-up period; (2) Rate of canine retraction was measured manually on plaster study models; (3) Anchorage loss was not measured; (4) The effect of different number and repetition of MOPs was not evaluated.

## CONCLUSIONS

1. Micro-osteoperforations temporarily increase the rate of tooth movement (first four weeks).
2. There was no difference in the rate of tooth movement from the fourth to the sixteenth week.
3. There was an increased level of biomarkers (ALP and acid phosphatase) in MOPs group, as compared to control.
